# Field efficacy of two atoxigenic biocontrol products for mitigation of aflatoxin contamination in maize and groundnut in Ghana

**DOI:** 10.1016/j.biocontrol.2020.104351

**Published:** 2020-11

**Authors:** Daniel Agbetiameh, Alejandro Ortega-Beltran, Richard T. Awuah, Joseph Atehnkeng, Abuelgasim Elzein, Peter J. Cotty, Ranajit Bandyopadhyay

**Affiliations:** a International Institute of Tropical Agriculture (IITA), Ibadan 200001, Nigeria; b Department of Crop and Soil Sciences, Kwame Nkrumah University of Science and Technology, Kumasi, Ghana; c United States Department of Agriculture – Agricultural Research Service, Tucson, AZ 85721, USA; d School of Food Science and Engineering, Ocean University of China, Qingdao, China

**Keywords:** Aflatoxin, Biocontrol, Efficacy, Maize, Groundnut, Ghana

## Abstract

Biological control is one of the recommended methods for aflatoxin mitigation. Biocontrol products must be developed, and their efficacy demonstrated before widespread use. Efficacy of two aflatoxin biocontrol products, Aflasafe GH01 and Aflasafe GH02, were evaluated in 800 maize and groundnut farmers’ fields during 2015 and 2016 in the Ashanti, Brong Ahafo, Northern, Upper East, and Upper West regions of Ghana. Both products were developed after an extensive examination of fungi associated with maize and groundnut in Ghana. Each product contains as active ingredient fungi four *Aspergillus flavus* isolates belonging to atoxigenic African *Aspergillus* Vegetative Compatibility Groups (AAVs) widely distributed across Ghana. An untreated field was maintained for each treated field to determine product efficacy. Proportions of atoxigenic AAVs composing each product were assessed in soils before product application, and soils and grains at harvest. Significant (*P* < 0.05) displacement of toxigenic fungi occurred in both crops during both years, in all five regions. Biocontrol-treated crops consistently had significantly (*P* < 0.05) less aflatoxins (range = 76% to 100% less; average = 99% less) than untreated crops. Results indicate that both biocontrol products are highly efficient, cost-effective, environmentally safe tools for aflatoxin mitigation. Most crops from treated fields could have been sold in both local and international food and feed premium markets. Adoption and use of biocontrol products have the potential to improve the health of Ghanaians, and both income and trade opportunities of farmers, aggregators, distributors, and traders.

## Highlights

• Efficacy of 2 aflatoxin biocontrol products reported across crops, years and zones.
• Aflatoxin reduced by 99% in 800 maize and groundnut farmers’ fields in Ghana.
• Results are the most consistent and highest aflatoxin reductions reported to date.
• Displacement of toxigenic fungi by the active ingredients caused the reductions.
• Having both products offers greater versatility to modulate *Aspergillus* communities.

## 1 Introduction

Contamination of key staple and/or cash crops by toxic fungal metabolites, particularly aflatoxins, is an issue of significant public health and economic concern (Wu, 2015, JECFA, 2018). Although aflatoxins are produced by several *Aspergillus* species, *Aspergillus flavus* is the major aflatoxin producer (Klich, 2007). A wide range of crops including maize (*Zea mays* L.), groundnut (*Arachis hypogaea* L.), cottonseed (*Gossypium* spp.), pistachio (*Pistacia vera* L.), and almond (*Prunus dulcis* Mill.) are susceptible to infection by aflatoxin-producing fungi and subsequent aflatoxin contamination both in the field and after harvest (Cotty and Jaime-Garcia, 2007). Prevalence of aflatoxins is often high in crops grown in warm areas and exacerbated under conditions of drought and elevated temperatures (Cotty and Jaime-Garcia, 2007, Hamidou et al., 2014). With current climate change trends, the scope and prevalence of crop aflatoxin contamination is expected to increase worldwide (Battilani et al., 2016).

Efforts to protect consumers from adverse health effects of aflatoxins have resulted in several nations promulgating and enforcing standards to limit aflatoxin levels in foods and feeds (GSA, 2001, GSA, 2013, van Egmond et al., 2007, JECFA, 2018). However, lack of global harmonization of these standards have become a barrier to trade and restricts the competitiveness of commodities from countries with more relaxed standards to those with more stringent regulatory limits (Rios and Jaffee, 2008). Consequently, several exporting nations, including Ghana, have lost both access to premium European markets and huge trade revenues annually as a result of non-conformance to strictly monitored aflatoxin standards set by importing nations (Wu, 2004, Rios and Jaffee, 2008, Dzirasah, 2015). Similarly, losses in production revenue are incurred by poultry and livestock industries because of reduced productivity and increased mortality when animals are fed with aflatoxin contaminated feeds (Atherstone et al., 2016).

In Ghana, where crop aflatoxin contamination is perennial and dietary staples rely primarily on single cereals such as maize, chronic aflatoxin exposure and its consequent adverse health effects are rampant (Shuaib et al., 2012, Jolly et al., 2013, Afum et al., 2016, Kumi et al., 2016). Public health and economic consequences of crop aflatoxin contamination and human/animal exposure are numerous (Coulibaly et al., 2009, Wu, 2015, JECFA, 2018). Unfortunately, due to the stealthy nature of aflatoxins, many stakeholders in crop value chains, including producers and consumers, are not aware of the potential health and economic impacts posed by crop contamination (Awuah et al., 2009).

Aflatoxins are produced by diverse assemblages of fungi belonging to *Aspergillus* section Flavi (Frisvad et al., 2019). The most frequently implicated aflatoxin-producing species, *A. flavus* (Klich, 2007), is composed of the L and S morphotypes (Cotty, 1989). The L morphotype produces fewer, larger sclerotia (avg. dia > 400 μm), numerous conidia, and variable levels of B aflatoxins (Cotty, 1989). Some L morphotype genotypes lack the ability to produce aflatoxins (i.e., are atoxigenic) due to deletions, inversions, or genetic defects in one or more of the aflatoxin biosynthesis genes (Adhikari et al., 2016). The S morphotype, on the other hand, produces numerous small sclerotia (avg. dia < 400 μm), few conidia, and consistently high B aflatoxin levels. Worldwide, several morphologically similar but phylogenetically distinct fungi resembling the *A. flavus* S morphotype have been detected with some of them producing copious amounts of both B and G aflatoxins (Probst et al., 2014, Singh and Cotty, 2019). In West Africa, fungi with S morphotype producing both B and G aflatoxins are relatively common and have been known as unnamed taxon S_BG_ (Cardwell and Cotty, 2002, Atehnkeng et al., 2008, Probst et al., 2014). The unknown taxon S_BG_ fungi may be any of the recently described species *A. aflatoxiformans*, *A. austwickii*, *A. cerealis*, or *A. minisclerotigenes* (Pildain et al., 2008, Frisvad et al., 2019). Here we refer as S_BG_ strains to all fungi with S morphotype producing both B and G aflatoxins. *Aspergillus* species and morphotypes can be further subdivided into vegetative compatibility groups (VCGs). Members of a VCG descend from the same clonal lineage and therefore are isolated subpopulations (Grubisha and Cotty, 2010, Grubisha and Cotty, 2015).

Crop infection by toxigenic fungi can be prevented but once crops become contaminated, the toxin cannot be completely removed (Grenier et al., 2014). Consequently, several technologies that limit fungal infection of susceptible crops and prevent further accumulation of toxins in both on-farm and during pre- and postharvest stages have been recommended (Seetha et al., 2017, Ojiambo et al., 2018, Mahuku et al., 2019, Pandey et al., 2019). An effective innovation is biocontrol through the use of native atoxigenic *A. flavus* VCGs to displace toxigenic fungi from the crop environment. Biocontrol reduces preharvest crop aflatoxin contamination generally to safe levels with a carry-over effect that provides protection in storage (Bandyopadhyay et al., 2019, Ezekiel et al., 2019, Senghor et al., 2020). By lowering aflatoxin in food, biocontrol susbstantially reduces human/animal exposure to these noxious toxins.

Use of atoxigenic fungi aims to reshape the resident fungal community structure, typically dominated by aflatoxin producers, in favour of one with less aflatoxin-producing ability. Atoxigenic isolates of *A. flavus* endemic to specific regions have been identified and/or evaluated for their potential deployment in aflatoxin biocontrol programs on target crops in the same region (Cotty, 1989, Atehnkeng et al., 2008, Abbas et al., 2011, Probst et al., 2011, Alaniz Zanon et al., 2013, Wei et al., 2014, Mauro et al., 2015, Weaver et al., 2015, Ortega-Beltran et al., 2016, Molo et al., 2019, Savi et al., 2020). Similarly, eight native *A. flavus* isolates belonging to diverse atoxigenic African *Aspergillus flavus* VCGs (AAVs) with superior abilities to displace aflatoxin producers and move to crops were selected among 847 atoxigenic *A. flavus* isolates recovered from maize and groundnut grown in Ghana (Agbetiameh et al., 2019). Those selected atoxigenic isolates are potential agents for aflatoxin biocontrol in maize and groundnut in Ghana. However, their efficacies as active ingredient fungi in biocontrol formulations under multiple field conditions, during multiple years require further evaluation and validation.

In this study, the efficacy of two biocontrol products, named Aflasafe GH01 and Aflasafe GH02, each formulated with a combination of four atoxigenic AAVs native to Ghana as active ingredient fungi were simultaneously but independently evaluated for their efficacy in preventing aflatoxin contamination. Evaluations were conducted over a two-year period under farmer-field conditions on hundreds of maize and groundnut fields across three agroecozones (AEZs) in Ghana. Results from the efficacy studies indicate that both biocontrol products are highly effective in reducing pre-harvest aflatoxin contamination in maize and groundnut across all three AEZs. This is the first report of two aflatoxin biocontrol products evaluated simultaneosly during two years, in multiple fields of multiple AEZ, in two crops.

## 2 Materials and methods

### 2.1 Selection of atoxigenic active ingredients

Based on a previous study on the relative adaptation to maize and groundnut cropping systems, frequency of occurrence, and competitive potential to move to crops and limit crop aflatoxin content in three AEZs in Ghana (Agbetiameh et al., 2019), eight superior atoxigenic AAVs were selected as active ingredient fungi for the formulation of two aflatoxin biocontrol products: Aflasafe GH01 and Aflasafe GH02. Each biocontrol product was composed of a blend of four AAVs, each represented by respective type isolates, as active ingredient fungi. The atoxigenic AAVs in Aflasafe GH01 were also found to be widely distributed in several African nations and therefore formulated as West Africa-specific (regional) product (Islam et al., 2015). Those AAVs composing Aflasafe GH02 have been detected to be native only to Ghana as of now. The AAVs of the two biocontrol products developed for use in Ghana are maintained in the fungal culture collection of the Pathology and Mycotoxin laboratory of the International Institute of Tropical Agriculture (IITA), Ibadan-Nigeria (Table 1).

**Table 1:** Origin of type isolates of native atoxigenic African *Aspergillus flavus* Vegetative Compatibility Groups (AAVs) used as active ingredients in biocontrol products developed for aflatoxin mitigation in Ghana.

Product	AAV	AEZ^a^	Region	District	Latitude	Longitude
Aflasafe GH01	GHG079-4	DS	Brong Ahafo	Atebubu-Amanten	07°46.219′ N	00°58.616′ W
	GHG083-4	DS	Brong Ahafo	Atebubu-Amanten	07°45.961′ N	00°58.944′ W
	GHG321-2	SGS	Upper East	Nabdam	10°48.778′ N	00°45.139′ W
	GHM174-1	HF	Brong Ahafo	Wenchi	07°47.552′ N	02°10.846′ W
Aflasafe GH02	GHM001-5	DS	Eastern	Nsawam-Adoagyire	05°48.294′ N	00°20.649′ W
	GHM109-4	HF	Ashanti	Ejura-Sekyedumase	07°19.497′ N	01°25.715′ W
	GHM287-10	SGS	Upper West	Wa West	09°59.155′ N	02°34.767′ W
	GHM511-3	DS	Volta	Central Tongu	06°04.776′ N	00°34.953′ E

a AEZ: Agroecological zone in which the isolate was recovered. DS, Derived Savanna; SGS, Southern Guinea Savanna; HF, Humid Forest.

### 2.2 Formulation of biocontrol products

Each biocontrol product was composed of roasted, sterile sorghum grains as delivery carrier coated with a conidial suspension of a mixture of the type isolates of the four atoxigenic AAV active ingredients with the aid of a polymer. A blue food colorant was added to differentiate the product from regular sorghum (Atehnkeng et al., 2014). Conidia of the atoxigenic AAVs were obtained from 5-day-old cultures grown on 5–2 agar [(5% V-8 juice (Campbell Soup Company, Camden, NJ), 2% Bacto-agar (Difco Laboratories Inc., Detroit, MI), pH 6.0)] at 31 °C in the dark (Cotty, 1989). Spores were dislodged and suspended in 0.1% TWEEN 80®. Suspensions were adjusted to 10^6^ conidia/ml using a turbidimeter using a nephelometric turbidity Unit (NTU) *vs* colony-forming unit (CFU) standard curve (*y* = 49,937*x*; *x*  = NTU, *y* = spores/ml) (Atehnkeng et al., 2014). The products were formulated using a seed coater (Model AT500, USC™ LLC, Sabetha, KS, US) calibrated to coat 1 kg sterile sorghum grains with a suspension containing 10 ml of 10^6^ conidia/ml, 10 ml sterile distilled water, 1.5 ml of polymer (Sentry™, Precision Laboratories, Waukegan, IL, US) and 2 ml of blue food colorant (Prism™, Milliken and Company, Spartanburg, SC, US). Following phytosanitary certification by the Nigeria Agricultural Quarantine Service (NAQS) and the issuance of import permit by Ghana’s Environmental Protection Agency (EPA), the products were transported to Ghana for evaluation of their potential to reduce aflatoxin contamination in farmer-field trials.

### 2.3 Quality tests of the biocontrol products

For each product, a 100 g sample of formulated product was taken from every batch of 100 kg of product as described by Senghor et al. (2020). Briefly, from each 100 g sample, 24 grains of formulated product were randomly selected and placed in a 24-well plate, one grain per well. Spaces outside and between wells were filled with 12 ml sterile distilled water. Plates were placed inside a polyethylene bag containing a damp sterile paper towel. Subsequently, the bags were closed and incubated at 31 °C for 7 d. Microbial growth on grains were visually inspected and numbers of grains i) germinating, ii) with *A. flavus* growth, iii) with other fungal or bacterial growth, and iv) with fluffy mycelial growth causing reduced sporulation were recorded. The number of spores produced per g of product were calculated in three arbitrarily selected pairs of grains and were quantified with a turbidimeter as above.

The number of spores on the formulated product were calculated in each batch by mixing 1 g of product with 10 ml sterile distilled water in 40 ml vials and allowing to sit in a benchtop for 10 min. Vials were then vortexed for 30 sec. The spore washes were diluted up to 10^-4^ and aliquots of 100 µl were plated on 2% Bacto-agar plates. Plates were incubated at 31 °C and at the end of the incubation period (3 d) the number of CFU was recorded. In addition, the identity of the active ingredient fungi on each formulated product was verified using vegetative compatibility analysis (VCA) (Agbetiameh et al., 2019, Senghor et al., 2020). Nitrate non-utilizing (*nit*) auxotrophs were generated for 25 recovered *A*. *flavus* isolates from the spore washings from each batch (Grubisha and Cotty, 2010). Briefly, 20 µl spore suspension of each isolate was seeded into a well at the center of a plate containing Selection medium (Czapek-dox broth, 25 g/l KClO_3_, 10 ml rose Bengal, 2% Bacto-agar, pH 7.0). Seeded plates were incubated at 31 °C for 7 to 30 d. Spontaneous auxotrophic sectors were transferred to a purification medium (Czapek-dox broth, 15 g/l KClO_3_, 2% Bacto-agar, pH 6.5) for 3 d to clean up and *nit* mutant stabilization. A mutant sector was subsequently transferred onto 5-2 agar for 5 d at 31 °C. Agar plugs of sporulating mutants (3 mm dia) were stored in 4 ml glass vials containing 2 ml sterile distilled water for use in complementation assays. Assignment of mutants of isolates to an AAV was based on pairing the isolate auxotroph with complementary tester auxotrophs of each atoxigenic AAV (Grubisha and Cotty, 2010). A single complementation test was performed on a starch agar plate (36 g dextrose, 3 g NaNO_3_, 2% Bacto-agar, 2% soluble starch, pH 6.0) (Cotty and Taylor, 2003) where three wells (3 mm dia, 1 cm apart) were made in a triangular pattern at the center of the plate. Two wells were each seeded with 10 µl of either of the tester pair while the third well was seeded with the isolate auxotroph being characterized. Plates were incubated for up to 10 d at 31 °C. Auxotrophs forming a stable heterokaryon with one or both tester auxotrophs of an atoxigenic AAV were assigned to that AAV.

### 2.4 Site and field selection

**Fig. 1:**
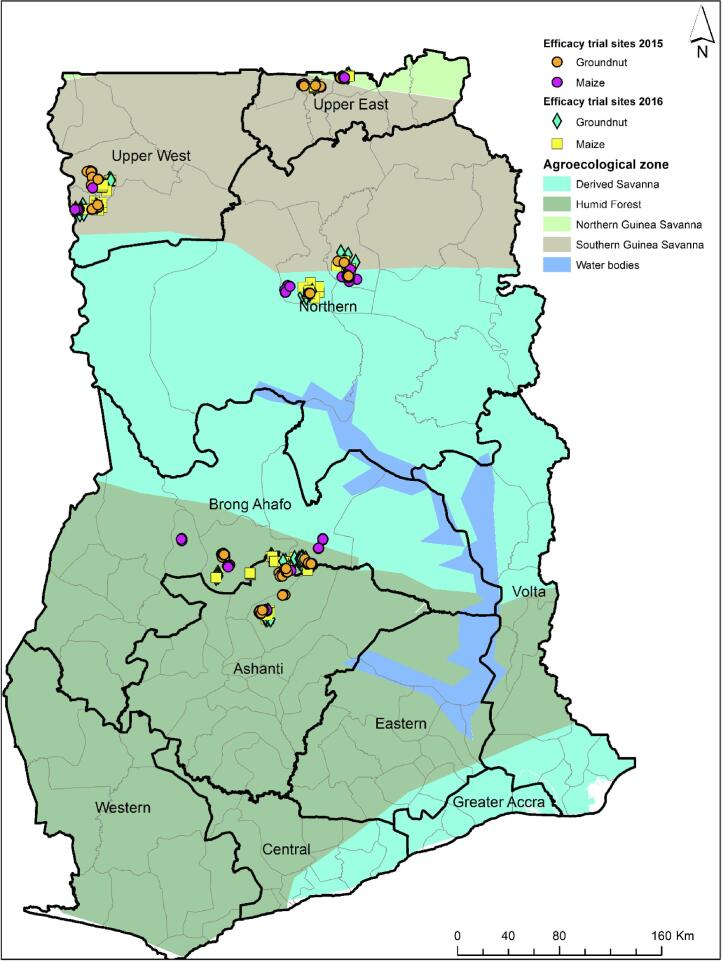
Map of Ghana indicating regions where efficacies of two biocontrol products were tested for aflatoxin mitigation in maize and groundnut during 2015 and 2016 cropping seasons.

Two districts each from five major maize and/or groundnut producing areas in Ghana known for high aflatoxin contamination events (MoFA, 2011, Sugri et al., 2015, Agbetiameh et al., 2018) were selected (Fig. 1). Stakeholders in the maize and groundnut value chains composed mainly of farmers and Agricultural Extension Agents (AEAs) of the Ministry of Food and Agriculture (MoFA) were sensitized and trained on the basics of crop aflatoxin contamination and its management including use of biocontrol products. Farmer and farmer-field selection was done in collaboration with respective District Department of Agriculture officials of MoFA. Each biocontrol product was evaluated in 200 maize and 200 groundnut farmer-fields in each of the 2015 and 2016 cropping seasons. The crops were grown by farmers following prevalent field and crop management practices in their respective areas without any other special intervention. An untreated field of the same crop, separated by 25 m to 200 m from treated fields, served as paired field for each treated field. Field size ranged from 0.5 to 2 ha. In the Middle Belt (Ashanti and Brong Ahafo regions), where two cropping seasons (major and minor) occur (Agbetiameh et al., 2018), field evaluations were conducted during the minor season in 2015 and the major season in 2016.

### 2.5 Product application and sample collection

**Fig. 2:**
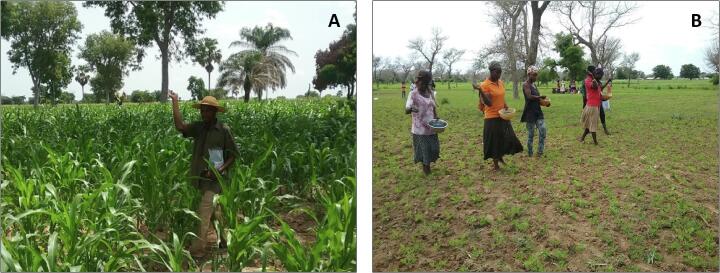
Farmers applying biocontrol products for aflatoxin mitigation in A) maize and B) groundnut fields in Ghana.

Products were broadcasted by hand on the soil surface 35 to 40 d after planting at a rate of 10 kg/ha as described by Agbetiameh et al. (2019) (Fig. 2). This time corresponded with 2–3 weeks before crop flowering. Farmers were advised to finalize agronomic operations before treatment and reduce movement in the field for about 7 to 10 d after treatment so that the product remained on the soil surface. Prior to product application, sub-samples of topsoil (~2 cm depth) were taken randomly from 50 different spots to compose a sample of about 150 g for each treated and corresponding untreated field. Similarly, soil samples were collected at harvest. Grains, comprising 30 maize ears and approximately 1 kg of groundnut (in-shell), were randomly collected at harvest from both treated and untreated fields. All crop and soil samples were sent to IITA-Ibadan under appropriate export/import permits for aflatoxin and microbiological analyses.

### 2.6 Aflatoxin quantification

Aflatoxin content in maize and groundnut collected at harvest from treated and untreated fields was determined using thin layer chromatography and quantified with a scanning densitometer as described by Agbetiameh et al. (2018). Grains were manually shelled and a half portion (500 g) was milled using a laboratory blender (Waring Commercial, Springfield, MO) for 1 min in a 1 L stainless steel blending jar (MC-2). Milled samples were stored at 4 °C prior to aflatoxin and microbial analyses. The blending jar was washed between samples with 80% ethanol to prevent microbial and aflatoxin cross contamination. Briefly, aflatoxins were extracted from maize by combining 20 g ground sample with 100 ml 70% methanol (Atehnkeng et al., 2008). For groundnut, 20 g ground sample were combined with 100 ml 80% methanol (Cole and Dorner, 1993). Suspensions were shaken on a Roto-Shake Genie (Scientific Industries, Bohemia, NY) for 30 min at 400 rpm and filtered through Whatman No. 1 filter paper (Whatman International ltd., Maidstone, England). Filtrates were collected in 250 ml separatory funnels, combined with 100 ml distilled water, and extracted twice with 25 ml methylene chloride. The methylene chloride phase was filtered through a bed of 25 g anhydrous sodium sulphate contained in fluted Whatman No. 4 filter paper, combined, and evaporated to dryness in a fume hood (Cotty and Cardwell, 1999). Residues were dissolved in 1 ml methylene chloride and subjected to scanning densitometry as described by Agbetiameh et al. (2018). The limit of quantification was 1 ppb.

### 2.7 Mycoflora analysis in soils and grains

Densities and composition of communities of *Aspergillus* section Flavi were determined in soil prior to application of the biocontrol products, soil at harvest, and grains at harvest. Briefly, soil samples were dried in a forced air oven (50 °C, 48 h), aseptically pulverized and sieved through 2 mm wire mesh to remove gravels and large particles. *Aspergillus* section Flavi fungi in soils and grains were isolated using dilution plate technique on modified rose Bengal Agar (MRBA) as described by Atehnkeng et al. (2014). Plates were incubated at 31 °C in the dark for 3 d. Incidences of *Aspergillus* spp. in soils and grains were calculated as CFU per g of sample. From each sample, 16 discrete *Aspergillus* colonies were sub-cultured on 5–2 agar at 31 °C for 7 d and subsequently assigned to their corresponding species based on macroscopic and microscopic characteristics (Pitt and Hocking, 2009). Sporulating cultures of each isolate were saved as agar plugs in 4 ml vials containing 2 ml sterile distilled water until further characterization.

### 2.8 Vegetative compatibility analyses

Proportions of isolates composing atoxigenic AAV active ingredients of each biocontrol product were determined within *A. flavus* populations recovered from all substrates, using VCA as described above. On an average, 12 isolates from each sample were used for VCA. A total of 57,400 VCA were conducted during both years. Auxotrophs forming stable heterokaryons with one or both tester auxotrophs of an atoxigenic AAV were assigned to that AAV and were considered to be the applied atoxigenic AAV.

### 2.9 Data analysis

Data for all response variables, including aflatoxin levels in grains, CFU/g, incidence of species and strains of *Aspergillus* section Flavi, and frequencies of atoxigenic AAVs, were log-transformed, using the equation [*y* = log_10_(response variable + 1)] to normalize the variance prior to analysis. All data was analyzed separately using the TTEST procedure of SAS (version 9.4, SAS Institute Inc., Cary, NC) by regions. Differences in means in all response variables between treated and untreated fields were separated, using Student’s *t*-test (α = 0.05).

## 3 Results

### 3.1 Quality test check for formulated products

All 30 batches of each product were sampled and assayed, and each grain of both products was colonized only by *A. flavus*. Other microorganisms were not detected. All isolates recovered from the product were identified as belonging to the VCG of one constituent active ingredient AAV of the respective biocontrol product. Other VCGs of *A. flavus* were not detected in any batch. In each biocontrol product, each of the four active ingredient AAVs was found on 25% ±3 carrier grains of the examined batches. The spore yield per gram of product following incubation in 24-well plates for 7 d ranged from 560 × 10^9^ to 650 × 10^9^ conidia. The washings from each gram of the products contained on an average, 3,500 ± 300 CFU coated on the surface of the sorghum grains.

### 3.2 Aflatoxin levels in treated and untreated maize and groundnut grains

In both years across all regions aflatoxin concentrations were below the limit of detection (1 ppb) at harvest in most groundnut and maize treated with either Aflasafe GH01 or Aflasafe GH02 (Table 2, Table 3). On an average, aflatoxin concentration compared to untreated fields was less in treated fields by 98.6% for Aflasafe GH01 and 99.5% for Aflasafe GH02. Aflatoxins were not detected in > 95% of the maize treated with Aflasafe GH02 during the two successive years with only few maize samples from Upper West in 2016 containing even low aflatoxin content (avg. = 6 ppb total aflatoxins). Aflatoxins were only detected (avg. range = 0.1 to 14.0 ppb total aflatoxins; Table 2) in groundnut treated with Aflasafe GH01 in two regions each year. On the other hand, aflatoxin levels in maize and groundnut from untreated fields varied markedly in both years. Average total aflatoxin content of Aflasafe GH01 untreated crops ranged from 2.1 to 301 ppb in maize and from 2.8 to 939 ppb in groundnut (Table 2). In general, higher aflatoxin content was detected in untreated crops during 2016 compared to 2015, particularly in groundnut in Brong Ahafo (DS), Ashanti (HF) and Upper West (SGS) (Table 2). Untreated maize in Brong Ahafo (DS) and Ashanti (HF) was relatively less contaminated during the two years (avg. range = 2.4 to 8.3 ppb).

**Table 2:** Aflatoxin levels in maize and groundnut grains from Aflasafe GH01-treated and untreated fields across three agroecological zones (AEZs) in Ghana in 2015 and 2016.

AEZ^a^	Region	Treatment^b^	N^c^	Aflatoxin concentration (ppb^d^)										
				Maize						Groundnut				
				2015			2016			2015			2016	
				Mean	% Red^e^		Mean	% Red		Mean	% Red		Mean	% Red
DS	Brong Ahafo	Treated	20	0.0*	100		0.0*	100		0.0*	100		0.0*	100
		Untreated	20	7.3			21.0			40.0			26.0	
	Northern	Treated	40	0.0*	100		0.0*	100		0.0*	100		0.0*	100
		Untreated	40	98.0			238.0			2.8			199.0	
HF	Ashanti	Treated	40	0.0*	100		0.0*	100		0.0*	100		14.0*	76
		Untreated	40	2.9			8.3			293.0			59.0	
	Brong Ahafo	Treated	20	0.0*	100		0.0*	100		0.0	100		2.2*	98
		Untreated	20	4.5			2.4			2.2			135.0	
SGS	Upper East	Treated	40	0.0*	100		0.0*	100		0.1*	99		0.0*	100
		Untreated	40	4.7			122.0			13.0			200.0	
	Upper West	Treated	40	0.0*	100		6.0*	98		0.3*	99		0.0*	100
		Untreated	40	6.3			301.0			53.0			939.0	

a DS, Derived Savanna; HF, Humid Forest; SGS, Southern Guinea Savanna.

b Treated refers to fields to which Aflasafe GH01 was applied at the rate of 10 kg/ha. Untreated were nearby fields separated by at least 25 m from corresponding treated field in which no biocontrol product was applied.

c Indicates total number of maize and groundnut fields treated with Aflasafe GH01 and their corresponding untreated fields in each year.

d Mean aflatoxin values correspond to total aflatoxin concentrations. An asterisk (*) indicates significant (*P* < 0.05) differences in aflatoxin levels between treated and untreated grains in each crop/region (Student’s *t*-test; α = 0.05).

e % reduction = {(mean of untreated fields – mean of treated fields) / mean of untreated fields} * 100

**Table 3:** Aflatoxin levels in maize and groundnut from Aflasafe GH02-treated and untreated fields across three agroecological zones (AEZs) in Ghana in 2015 and 2016.

AEZ^a^	Region	Treatment^b^	N^c^	Aflatoxin concentration (ppb^d^)										
				Maize						Groundnut				
				2015			2016			2015			2016	
				Mean	% Red^e^		Mean	% Red		Mean	% Red		Mean	% Red
DS	Brong Ahafo	Treated	20	0.0*	100		0.0*	100		0.0*	100		0.0*	100
		Untreated	20	2.9			1.6			2.7			4.5	
	Northern	Treated	40	0.0*	100		0.0*	100		0.0*	100		0.0*	100
		Untreated	40	5.9			70.0			2.3			50.0	
														
HF	Ashanti	Treated	40	1.0*	80		0.3*	99		0.0*	100		2.5*	99
		Untreated	40	4.9			28.0			55.0			722.0	
	Brong Ahafo	Treated	20	0.0*	100		0.0*	100		0.0*	100		2.4*	99
		Untreated	20	2.0			3.5*			8.4			557.0	
														
SGS	Upper East	Treated	40	0.0*	100		0.0*	100		0.1*	98		0.0*	100
		Untreated	40	5.0			238.0			4.0			8.1	
	Upper West	Treated	40	0.0*	100		1.7*	99		0.0*	100		0.0*	100
		Untreated	40	8.2			325.0			0.8			14.0	

a DS, Derived Savanna; HF, Humid Forest; SGS, Southern Guinea Savanna.

b Treated refers to fields to which Aflasafe GH02 was applied at the rate of 10 kg/ha. Untreated were nearby fields separated by at least 25 m from corresponding treated field in which no biocontrol product was applied.

c Indicates total number of maize and groundnut fields treated with Aflasafe GH01 and their corresponding untreated fields in each year.

d Mean aflatoxin values correspond to total aflatoxin concentrations. An asterisk (*) indicates significant (*P* < 0.05) differences in aflatoxin levels between treated and untreated grains in each crop/region (Student’s *t*-test; α = 0.05).

e % reduction = {(mean of untreated fields - mean of treated fields) / mean of untreated fields} *100.

Treatment of crops with Aflasafe GH02 resulted in significantly (*P* < 0.05) less (80% to 100%) aflatoxins compared to untreated crops across all three AEZs (Table 3). In any given region, total aflatoxin concentration in Aflasafe GH02-treated crops did not exceed 2.5 ppb. Average total aflatoxin content of Aflasafe GH02 untreated crops ranged from 1.6 to 325 ppb in maize and from 0.8 to 722 ppb in groundnut (Table 3).

### 3.3 Community composition of Aspergillus section Flavi in soil and grains

In both years and across AEZs, fungal communities in soils prior to application of both biocontrol products were dominated (62.5 to 100%) by the *A. flavus* L morphotype. Incidences of S_BG_ strains, *A. parasiticus*, and *A. tamarii* were minor in both untreated and treated fields prior to treatment (Table 4, Table 5; Supplementary Table 1, Table 2).

**Table 4:** Combined frequencies and distribution of *Aspergillus* section Flavi in soils and grains from Aflasafe GH01-treated and untreated fields across three agroecological zones (AEZs) in Ghana.

Year	AEZ^a^	Treatment^b^	Frequencies of *Aspergillus* section *Flavi*^c^, ^d^ (%)													
			Soil before inoculation					Soil at harvest					Grains^e^			
			L	S_BG_	P	T		L	S_BG_	P	T		L	S_BG_	P	T
2015	DS	Treated	98.3	0.0	0.3	1.4		99.3*	0.0*	0.4	0.3		97.2	1.7	0.0	1.1
		Untreated	94.8	1.7	1.1	2.4		89.2	8.7	1.0	1.1		98.9	0.7	0.4	0.0
	HF	Treated	84.0	0.0	3.8	12.2*		95.8	0.4	0.0	3.8		98.3	1.7	0.0	0.0
		Untreated	87.0	3.4	6.8	2.8		87.2	0.4	5.6	6.8		100.0	0.0	0.0	0.0
	SGS	Treated	96.5	0.0	1.7	1.8		98.0	0.0	1.0	1.0		99.2	0.0	0.0	0.8
		Untreated	96.1	1.8	0.5	1.6		96.8	1.0	1.9	0.3		94.8	4.2	0.0	1.0
																
2016	DS	Treated	94.2	2.4	1.0	2.4		99.0	0.0	0.0	1.0		100.0	0.0	0.0	0.0
		Untreated	86.5	2.0	4.8	6.7		87.5	7.2	1.9	3.4		98.5	1.5	0.0	0.0
	HF	Treated	93.4	2.1	1.7	2.8		97.6	0.3	0.0	2.1		100.0	0.0	0.0	0.0
		Untreated	95.8	0.7	3.2	0.3		95.8	1.4	0.7	2.1		98.6	0.7	0.4	0.3
	SGS	Treated	90.6	3.2	3.1	3.1		100.0	0.0	0.0	0.0		97.5	1.3	0.0	1.2
		Untreated	88.1	3.8	0.6	7.5		96.3	0.6	0.6	2.5		87.5	1.3	10.6	0.6

a DS, Derived Savanna; HF, Humid Forest; SGS, Southern Guinea Savanna.

b Treated refers to fields to which Aflasafe GH01 was applied at the rate of 10 kg/ha. Untreated were nearby fields separated by at least 25 m from corresponding treated field in which no biocontrol product was applied.

c L = *A. flavus* L morphotype, S_BG_ = S_BG_ strains, P = *A. parasiticus,* T = *A. tamarii.*

d In each AEZ, species frequencies from treated samples with an asterisk (*) significantly differed from those found in corresponding untreated samples by Student’s *t*-test (α = 0.05).

e Values depict means for both maize and groundnut grains.

**Table 5:** Combined frequencies and distribution of *Aspergillus* section Flavi in soils and grains from Aflasafe GH02-treated and untreated fields across three agroecological zones (AEZs) in Ghana.

Year	AEZ^a^	Treatment^b^	Frequencies of *Aspergillus* section *Flavi*^c^, ^d^ (%)													
			Soil before inoculation					Soil at harvest					Grains^e^			
			L	S_BG_	P	T		L	S_BG_	P	T		L	S_BG_	P	T
2015	DS	Treated	92.7	0.0	5.6	1.7		97.2*	2.4	0.4	0.0		99.3	0.7	0.0	0.0
		Untreated	94.8	1.0	3.5	0.7		85.8	4.5	9.0	0.7		98.0	1.7	0.0	0.3
	HF	Treated	81.5	0.7	15.7	2.1		93.8	5.2	0.7	0.3		99.7*	0.3*	0.0	0.0
		Untreated	78.7	2.2	13.2	5.9		90.0	2.8	5.5	1.7		82.6	17.4	0.0	0.0
	SGS	Treated	98.2	0.0	0.0	1.8		98.8	0.9	0.0	0.3		100.0	0.0	0.0	0.0
		Untreated	96.4	0.8	0.8	2.0		96.7	1.2	0.7	1.4		99.5	0.0	0.0	0.5
																
2016	DS	Treated	94.6	0.5	0.0	4.9		100.0*	0.0	0.0	0.0		99.1	0.5	0.4	0.0
		Untreated	96.0	0.9	1.3	1.8		86.6	4.0	2.2	7.2		98.7	0.4	0.9	0.0
	HF	Treated	93.1	2.4	1.7	2.8		99.0	0.0	0.3	0.7		98.3	0.7	1.0	0.0
		Untreated	93.8	0.7	4.2	1.3		96.5	1.4	1.7	0.4		95.5	3.8	0.0	0.7
	SGS	Treated	95.6	1.3	0.6	2.5		100.0*	0.0	0.0	0.0		100.0	0.0	0.0	0.0
		Untreated	95.0	1.3	2.5	1.2		90.6	5.0	1.9	2.5		99.4	0.0	0.6	0.0

a DS, Derived Savanna; HF, Humid Forest; SGS, Southern Guinea Savanna.

b Treated refers to fields to which Aflasafe GH02 was applied at the rate of 10 kg/ha. Untreated were nearby fields separated by at least 25 m from corresponding treated field in which no biocontrol product was applied.

c L = *A. flavus* L morphotype, S_BG_ = S_BG_ strains, P = *A. parasiticus,* T = *A. tamarii.*

d In each AEZ, species frequencies from treated samples with an asterisk (*) significantly differed from those found in corresponding untreated samples by Student’s *t*-test (α = 0.05).

e Values depict means for both maize and groundnut grains.

In soils collected at harvest from both maize and groundnut fields across all AEZs, the application of the biocontrol products generally resulted in increased proportions of *A. flavus* L morphotype and reduced incidences of S_BG_ strains, *A. parasiticus*, and *A. tamarii* (Table 4, Table 5). For instance, incidences of L morphotype in maize field soils from Brong Ahafo (HF) increased from 71% before biocontrol application to 98% at harvest while *A. parasiticus* drastically decreased from 28% to 1% in the same time frame (Supplementary Table 2). Fungi from soils at harvest of untreated fields were, in most cases, composed of two or more *Aspergillus* section *Flavi* species/strains.

In grains, communities from treated fields were dominated by the L morphotype with 98% to 100% incidence (Table 4, Table 5; Supplementary Table 1, Table 2). Low proportions (up to 2%) of S_BG_ strains and/or *A. tamarii* constituted the remaining portion of the population. Compared to grains from untreated fields, significantly (*P* < 0.05) lower L morphotype incidences were observed in a few cases. In 2015 for instance, the mean incidence of L morphotype in grains from untreated fields in Ashanti (HF) (76.1%) was significantly (*P* < 0.05) lower than the mean incidence (100%) from corresponding treated fields (Supplementary Table 2). Conversely, higher proportions of S_BG_ strains, *A. parasiticus*, and *A. tamarii* were generally recovered from untreated grains in comparison to treated grains (Table 4, Table 5).

### 3.4 Densities of Aspergillus section Flavi in soils and grains

In both years, fungal densities varied in soils and grains across AEZs, regions, and treatments irrespective of biocontrol treatments (Table 6). Fungal densities were generally lower in soils prior to treatment and highest in grains at harvest. Densities of *Aspergillus* section Flavi ranged from 9 to 2,877 CFU/g in soils prior to application of either biocontrol product in both years and no significant (*P* > 0.05) differences were observed within treatments in any of the comparisons (Table 6). In soils at harvest, however, densities were generally higher in treated soils and ranged from 12 to 4,542 CFU/g across regions and years. Higher fungal densities were detected in grains at harvest, but these varied between treatments. In certain regions, fungal densities in grains from untreated fields were higher than in the corresponding treated fields while in others the opposite occurred. In 2015 for instance, grains treated with Aflasafe GH01 in Brong Ahafo (DS) had 84,671 CFU/g compared to 1,755 CFU/g in grains from corresponding untreated fields. In contrast, fungal densities in grains from treated fields were lower (180 CFU/g) than that from corresponding untreated fields (16,209 CFU/g) in the Northern region during 2015. Overall, densities in grains treated with Aflasafe GH01 ranged from 47 to 167,030 CFU/g while that from untreated fields ranged from 61 to 10^6^ CFU/g. In comparison to grains treated with Aflasafe GH01, fungal densities in Aflasafe GH02-treated grains were relatively higher and ranged from 119 to 861,243 CFU/g while those from paired untreated fields ranged from 129 to 1.4 × 10^6^ CFU/g (Table 6).

**Table 6:** Densities (colony-forming unit g^−1^) of *Aspergillus* section Flavi in soils and grains from Aflasafe GH01 and Aflasafe GH02 treated and untreated fields before biocontrol application and at harvest in five regions across three agroecological zones (AEZs) in Ghana.

AEZ^a^	Region	Treatment^b^	Aflasafe GH01^c^								Aflasafe GH02^c^						
			2015				2016				2015				2016		
			SB^d^	SH^e^	Grain^f^		SB	SH	Grain		SB	SH	Grain		SB	SH	Grain
DS	Brong Ahafo	Treated	69	872*	84,671*		20	132	226		58	4,542*	28,887		28	87	428
		Untreated	72	37	1,755		41	76	34,713		50	12	129		208	188	11,645
	Northern	Treated	422	841*	180		14	891	18,984*		350	632*	119		236	519	9,082
		Untreated	358	162	16,209		31	188	263,197		529	228	229		122	275	173,118
HF	Ashanti	Treated	161	896*	36,645		436	1,486	167,030		101	362*	29,862		41	528	861,243*
		Untreated	308	33	19,105		90	462	40,502		77	242	4,944		69	802	1.4*10^6^
	Brong Ahafo	Treated	57	2,425*	6,823		84	581	953		9	695*	27,395		195	2,314	1,687*
		Untreated	30	29	61		230	146	9,683		33	20	33,580		171	469	117,002
SGS	Upper East	Treated	734	215*	5,053*		19	1,131*	47*		1,127	669*	866*		84	1,314	502
		Untreated	2,877	131	27		64	81	420,035		639	41	2,512		38	66	5,187
	Upper West	Treated	834	415*	625		22	1,688*	22,828*		302	346*	2,123		846	2,693	22,429*
		Untreated	254	210	15,608		28	230	1.0*10^6^		644	23	58,455		46	143	323,524

a DS, Derived Savanna; HF, Humid Forest; SGS, Southern Guinea Savanna.

b Treated refers to fields to which an Aflasafe product was applied at the rate of 10 kg/ha. Untreated were nearby fields separated by at least 25 m from corresponding treated field in which no biocontrol product was applied.

c In each region, by individual year, CFU/g from treated samples with an asterisk (*) significantly differed from its corresponding untreated samples by Student’s *t*-test (α = 0.05).

d SB = Soil samples were collected before field inoculation.

e SH = Soil samples were collected at harvest.

f Values are means for both maize and groundnut grains sampled at harvest.

### 3.5 Recovery of atoxigenic biocontrol AAVs from soils and grains

Atoxigenic AAV active ingredients in biocontrol products were relatively common in soils across AEZs in both years prior to application of either biocontrol product (Table 7, Table 8). For instance, out of 720 L morphotype isolates recovered from maize soil prior to biocontrol application in 2015, 2% belonged to AAV active ingredients composing Aflasafe GH02 (Table 8). Following the application of both biocontrol products, frequencies of atoxigenic AAV active ingredients in most instances increased in soil and grains collected at harvest compared to the levels detected prior to application. Also, in most instances, the frequencies of AAV active ingredients in treated soils and grains at harvest were significantly (*P* < 0.05) higher than in corresponding untreated substrates in both years and across AEZs (Table 7, Table 8). In 2015 for example, frequencies of Aflasafe GH01 AAVs in maize grains from treated fields ranged from 54.2% in Ashanti (HF) to 80.6% in Brong Ahafo (HF) (Table 7). Those frequencies were significantly (*P* < 0.01) higher than in grains from corresponding untreated fields which ranged from 0% in Brong Ahafo (DS) to 13.9% in Northern region (DS) (Table 7). Similarly, significantly (*P* < 0.05 or *P* < 0.01) higher frequencies of atoxigenic biocontrol AAVs compared to untreated samples were recovered from groundnut soils and groundnut kernels from treated fields at harvest in most regions and across AEZs, in both years (Table 7, Table 8). However, in 4% and 23% of the soil at harvest and grain comparisons, respectively, there were no significant differences (*P* > 0.05) in frequencies of atoxigenic AAVs between treated and untreated samples (Table 7, Table 8). In untreated samples, incidences of AAV active ingredients reached up to 44% and 53% in soil at harvest and grains, respectively.

**Table 7:** Incidence (%) of atoxigenic African *Aspergillus flavus* Vegetative Compatibility Groups (AAVs) composing Aflasafe GH01 in soils and grains in five regions across three agroecological zones (AEZs) in Ghana.

			Incidence (%)														
AEZ^a^	Region	Treatment^b^	Maize								Groundnut						
			2015				2016				2015				2016		
			SB^c^	SH^d^	Grain^e^		SB	SH	Grain		SB	SH	Grain		SB	SH	Grain

DS	Brong Ahafo	Treated	0.0	83.3^**^	75.0^**^		0.0	77.8^**^	72.2^**^		2.8	74.9^**^	58.3		2.8	80.6^**^	69.4^**^
		Untreated	2.8	3.7	0.0		2.8	2.8	2.8		0.0	0.0	52.8		2.8	2.8	0.0
	Northern	Treated	0.0	73.6^**^	64.8^**^		1.4	81.9*	87.5*		0.0	61.1^**^	63.9^**^		8.3	79.2^**^	66.7^**^
		Untreated	4.2	0.0	13.9		0.0	44.4	36.1		2.8	1.4	0.0		0.0	8.3	0.0
HF	Ashanti	Treated	0.0	66.1^**^	54.2^**^		0.0	84.7^**^	87.5^**^		2.8	68.1^**^	76.4^**^		5.5	81.9^**^	56.9^**^
		Untreated	0.0	4.9	8.3		5.5	19.4	22.2		0.0	0.0	9.7		0.0	9.7	5.5
	Brong Ahafo	Treated	0.0	94.4^**^	80.6^**^		2.8	79.8^**^	75.0*		0.0	83.3^**^	77.8		0.0	72.2^**^	66.7^**^
		Untreated	0.0	0.0	8.3		0.0	8.3	27.8		0.0	2.8	55.6		0.0	0.0	2.8
SGS	Upper East	Treated	0.0	58.3^**^	79.2^**^		0.0	77.8^**^	77.8^**^		0.0	59.7^**^	70.8^**^		0.0	91.7^**^	62.5
		Untreated	0.0	4.2	11.1		0.0	0.0	2.8		0.0	0.0	13.9		0.0	29.2	37.5
	Upper West	Treated	0.0	72.2^**^	69.4^**^		0.0	79.2^**^	75.0^**^		0.0	58.3^**^	62.5^**^		0.0	72.2^**^	66.7^**^
		Untreated	2.8	1.4	4.2		2.1	10.4	14.6		0.0	2.8	5.6		0.0	2.8	0.0

Significance levels, * (*P <* 0.05) and ** (*P <* 0.01) for testing the differences between treatment means within regions based on Student’s *t*-test (α = 0.05).

a DS, Derived Savanna; HF, Humid Forest; SGS, Southern Guinea Savanna.

b Treated refers to fields to which Aflasafe GH01 was applied at the rate of 10 kg/ha. Untreated were nearby fields separated by at least 25 m from corresponding treated field in which no biocontrol product was applied.

c SB = Soil collected from fields prior to application of Aflasafe GH01.

d SH = Soil collected at harvest.

e Grain = Maize or groundnut kernels at harvest.

**Table 8:** Incidence (%) of atoxigenic African *Aspergillus flavus* vegetative compatibility groups (AAVs) composing Aflasafe GH02 in soils and grains in five regions across three agroecological zones (AEZs) in Ghana.

AEZ^a^	Region	Treatment^b^	Incidence (%)														
			Maize								Groundnut						

			2015				2016				2015				2016		
			SB^c^	SH^d^	Grain^e^		SB	SH	Grain		SB	SH	Grain		SB	SH	Grain

DS	Brong Ahafo	Treated	5.6	75.0^**^	55.6*		0.0	83.3^**^	86.1*		5.6	69.4^**^	83.3^**^		0.0	83.3^**^	75.0^**^
		Untreated	5.6	8.3	16.7		2.8	2.8	38.9		5.6	0.0	0.0		0.0	0.0	11.1
	Northern	Treated	0.0	81.9^**^	61.1*		0.0	81.9*	81.9^**^		0.0	81.9^**^	55.0^**^		8.3	79.2^**^	54.2*
		Untreated	0.0	6.9	25.5		1.4	0.0	2.8		0.0	0.0	9.7		0.0	4.2	0.0
HF	Ashanti	Treated	3.5	77.8*	63.9*		5.8	81.9^**^	84.7		1.4	75.5^**^	61.1^**^		1.4	79.2^**^	79.2^**^
		Untreated	4.2	26.4	31.9		11.1	19.4	56.9		2.8	8.3	1.4		1.4	13.9	11.1
	Brong Ahafo	Treated	0.0	72.2^**^	97.2*		2.8	75.0^**^	72.2*		0.0	77.8^**^	52.8^**^		5.5	75.0^**^	69.4^**^
		Untreated	0.0	0.0	66.7		16.7	16.7	30.5		0.0	9.4	0.0		0.0	0.0	0.0
SGS	Upper East	Treated	0.0	81.9^**^	69.4^**^		0.0	79.2^**^	79.2		0.0	76.8^**^	40.7*		0.0	83.3^**^	70.8^**^
		Untreated	2.8	0.0	18.1		4.2	0.0	54.2		0.0	0.0	11.1		0.0	0.0	0.0
	Upper West	Treated	1.4	73.6^**^	87.5^**^		0.0	80.5^**^	68.0^**^		8.3	56.9^**^	47.2*		0.0	79.2^**^	70.8^**^
		Untreated	1.4	2.8	16.9		0.0	9.7	6.9		1.4	0.0	6.9		0.0	5.5	4.2

Significance levels, *(*P <* 0.05) and ** (*P <* 0.01) for testing the differences between treatment means within regions based on Student’s *t*-test (α = 0.05).

a DS, Derived Savanna; HF, Humid Forest; SGS, Southern Guinea Savanna.

b Treated refers to fields to which Aflasafe GH02 was applied at the rate of 10 kg/ha. Untreated were nearby fields separated by at least 25 m from corresponding treated field in which no biocontrol product was applied.

c SB = Soil collected from fields prior to application of Aflasafe GH02.

d SH = Soil collected at harvest.

e Grain = Maize or groundnut kernels at harvest.

## 4 Discussion

In the current study, efficacies of two aflatoxin biocontrol products were evaluated in maize and groundnut across three AEZs of Ghana for two successive years. The active ingredient fungi in both biocontrol products clearly established themselves in the soil and displaced aflatoxin producers which resulted in undetectable or substantially reduced aflatoxin levels in crops from treated fields, compared to those from untreated fields. The two products were equally effective at displacing aflatoxin producers and reducing aflatoxin in crops (less than untreated by 98.6% for Aflasafe GH01 and 99.5% for Aflasafe GH02) even though the products contain different active ingredient AAVs. This demonstrates the robustness of atoxigenic strain-based biocontrol. The lowest observed aflatoxin reduction was 76% in the Ashanti Region in groundnut and even then, mean aflatoxin content was reduced to 14 ppb. In most cases, treated crops did not contain detectable aflatoxins, regardless of product used, area, and year. Aflatoxin reductions through use of atoxigenic fungi have been reported worldwide in research efforts aiming at improving health and wealth outcomes (Dorner, 2004, Dorner, 2010, Atehnkeng et al., 2014, Doster et al., 2014, Weaver et al., 2015, Alaniz Zanon et al., 2016, Mauro et al., 2018, Bandyopadhyay et al., 2019, Ezekiel et al., 2019, Savi et al., 2020, Senghor et al., 2020). However, results from the current study provide, to our knowledge, the most consistent and highest aflatoxin reductions of any biocontrol product tested to date with results based on a comprehensive 400 treated fields per product. Furthermore, in the cases where mean crop aflatoxin concentrations exceeded 200 ppb, at least 98% reductions in contamination were observed (Table 2, Table 3).

Active ingredients for the two Aflasafe biocontrol products tested herein were selected from initial field evaluations of three experimental products with a total of 12 distinct atoxigenic AAVs (Agbetiameh et al., 2019). Although all three experimental products were effective in reducing contamination, active ingredients of Aflasafe GH01 and GH02 were selected based on both extent of aflatoxin reductions and incidence from the treated crops in that previous study. This rigorous selection process allowed identification of AAV active ingredients that are competitive and adapted to Ghana’s agricultural environments. Results from the current study validate the former study and indicate that products utilizing the selected mixtures of atoxigenic AAVs are highly effective and potentially sustainable tools for reducing aflatoxin contamination of maize and groundnut throughout Ghana.

Incidences of specific members of *Aspergillus* section Flavi present in soils and grains varied across regions and AEZs in both years. This is consistent with previous observations which indicate that *Aspergillus* communities in agricultural fields consist of individuals with diverse morphological and phenotypic characteristics (Cotty et al., 1994, Cardwell and Cotty, 2002). Prior to application of biocontrol products, *A. flavus* L morphotype dominated section *Flavi* communities (incidence > 62%). High incidences of L morphotype in both maize and groundnut soils before biocontrol application was expected as this fungus is recognized as the most common colonizer of crop substrates (Dorner and Horn, 2007, Atehnkeng et al., 2014) and the L morphotype the dominant (93% incidence) section Flavi member associated with maize and groundnut in Ghana (Agbetiameh et al., 2018). Proportions of S_BG_ strains and *A. parasiticus* ranged from 0 to 5% and 0 to 34%, respectively, while that of *A. tamarii* ranged from 0 to 17% (Table 4, Table 5). Tools used in the current study could not differentiate among the S_BG_ species resident in West Africa, because placement of these fungi into species requires DNA based phylogenetic analyses (Singh and Cotty, 2019, Probst et al., 2014). However, all the S_BG_ species fungi resident in West Africa produce very high concentrations of aflatoxins in crops (Singh and Cotty, 2019, Cardwell and Cotty, 2002). Similarly, *A. parasiticus* is among the most consistently aflatoxigenic species of *Aspergillus* section Flavi (Probst et al., 2014, Kachapulula et al., 2017). The grain samples with highest proportions of *A. parasiticus* (i.e., the untreated for Aflasafe GH01 in the Upper West Region in 2016, Suppl. Table 1) were the most contaminated with aflatoxins for both crops across years.

Members of the atoxigenic AAVs composing either biocontrol product were relatively common in soils across AEZs prior to application. Indeed, natural widespread occurrence of these competitive atoxigenic AAVs in soils across Ghana was a criterion for their selection as potential active ingredients. These observations suggest that these atoxigenic AAVs of *A. flavus* have coexisted with aflatoxin producers in diverse AEZ in Ghana for long periods of time (Agbetiameh et al., 2018). However, natural frequencies of these AAVs are insufficient to reliably result in aflatoxin safe food and feeds. As a result of treatment early in the season, prior to formation of large *Aspergillus* section Flavi communities on the crops, the applied atoxigenic AAVs were able to multiply on the carrier sorghum grains and established as a founding population on the treated crop in lieu of other, potentially toxigenic, *Aspergilli* (Cotty and Mellon, 2006, Ortega-Beltran et al., 2019). Establishment of the AAVs was observed as increased frequencies of the atoxigenic AAVs on crops at harvest in treated fields. Similar reductions in frequencies of aflatoxin-producers and increases in AAV active ingredients of multi-AAV biocontrol products has been reported for AAVs selected either for use on maize, groundnut, and chili pepper in Nigeria (Atehnkeng et al., 2014, Bandyopadhyay et al., 2019, Ezekiel et al., 2019) or for use in Senegal (Senghor et al., 2020).

Tracking of the AAVs examined in the current study was a resource intensive and time-consuming activity that required completion of 57,400 vegetative compatibility analyses (VCA). The demonstrated movement of applied AAVs to grains of treated crops supports that the observed aflatoxin reductions (76 to 100% less; Table 2, Table 3) are attributable to alterations in the composition of crop associated *Aspergillus* communities. Aflatoxin content of crops was reduced by shifting the *Aspergillus* community composition so that aflatoxin-producers are far less common and the active ingredient atoxigenic AAVs are dominant. The results support that a primary mechanism of atoxigenic-strain based biocontrol is the reshaping the *Aspergillus* community structure in favor of the applied atoxigenic active ingredient fungi (Cotty et al., 1994, Cotty, 1994, Bandyopadhyay et al., 2016, Bandyopadhyay et al., 2019, Senghor et al., 2020).

Applications of atoxigenic biocontrol products can be made without increasing the combined densities of *A. flavus* and *A. parasiticus* on treated crops in the US and West Africa (Cotty, 1994, Mehl et al., 2012, Doster et al., 2014, Ezekiel et al., 2019). Overall, results from the current study agree with those previous studies. Fungal densities in soil and grains at harvest did not vary between treated and untreated fields in both years irrespective of biocontrol product. However, there were a few instances where this was not the case. For example, 48 times higher fungal densities were detected on grains from treated fields compared to untreated fields from Brong Ahafo in 2015 (Table 6), and in 2016, fungal densities on grains from treated fields were, in most instances, lower than those from untreated fields from the same region irrespective of product. Similar findings were recently reported from Nigeria (Bandyopadhyay et al., 2019) and Senegal (Senghor et al., 2020) where in most cases, fungal densities in grains from fields treated with biocontrol did not differ significantly from untreated grains. In a previous study, Atehnkeng et al. (2014) consistently detected higher fungal densities in grains from treated compared to untreated fields with an application rate of 40 kg/ha. In the current study, although products used were similar to those used in the Atehnkeng et al. study, both biocontrol products were applied at a 10 kg/ha and this resulted in the production of grains with both significantly less aflatoxin and similar quantities of fungal propagules compared to grains from untreated fields.

Spores of *A. flavus* are dispersed by wind, rain, and insects (Bock et al., 2004, Stephenson and Russell, 1974, Horn, 2003). Movement of atoxigenic biocontrol products into untreated areas can be significant (Bock et al., 2004, Cotty et al., 2008) and, as a result, in biocontrol studies significant distance is often maintained between treated and untreated fields to reduce inter-plot dispersal of atoxigenic AAVs (Agbetiameh et al., 2019, Bandyopadhyay et al., 2019, Senghor et al., 2020). Treated and untreated fields were separated by at least 25 m in the current study. However, this distance was insufficient to prevent relatively high frequencies of the biocontrol AAVs in soils and grains from some untreated fields in both years (Table 7, Table 8). For example, in 2015, natural occurrence of Aflasafe GH02 AAVs in untreated maize fields in Ashanti was 4%, which increased to 26% and 32% in untreated soil and grains at harvest, respectively (Table 8). This contributed to the low aflatoxin concentrations (avg. = 5 ppb) detected in samples from untreated fields in that region. Similar observations on movement of inoculum from treated plots to adjoining (20 m distance) untreated plots have been made in the US (Weaver and Abbas, 2019). This should be a particular concern for researchers using strip plots to compare aflatoxin reductions by atoxigenic biocontrol products. Untreated plots must be sufficiently separated from treated plots to avoid underestimation of efficacy. These observations also suggest AAVs will have positive influences not only on treated fields but also nearby untreated fields and supports the concept of area-wide application for effective management of aflatoxin contamination (Cotty et al., 2007). On the other hand, determining the appropriate maximum and minimum distance to conduct field efficacy trials of aflatoxin biocontrol products (and biocontrol products in general) deserves further investigation.

Newspaper headlines on poisonous aflatoxins in the maize-based staple Kenkey have repeatedly scared the populations of Ghana’s large cities (Awuah et al., 2009). Application of the biocontrol products examined in the current study across AEZ and years frequently (94%, n = 800) resulted in production of crops containing undetectable aflatoxin levels providing practical relief for this fear. The results from the field efficacy trials reported in the current study were used to prepare dossiers for registration of Aflasafe GH01 and Aflasafe GH02 with EPA-Ghana, the regulatory agency responsible for registering biological control agents. In June 2018, EPA-Ghana approved the unrestricted use of both products for aflatoxin mitigation in groundnut and maize throughout Ghana. In addition, EPA-Ghana allowed the use of both products in sorghum crops.

Ghana is the first nation for which a large array of atoxigenic germplasm (12 atoxigenic AAVs) has been tested extensively over multiple years, in multiple crops, and across multiple AEZ. Apart from area-wide applications for effectively reducing risks of aflatoxin contamination (Cotty et al., 2007), a potential strategy to further reduce the risk of contamination is to rotate mixtures of atoxigenic AAVs between seasons and crops to promote a more diverse, stable atoxigenic community with a large repertoire of adaptive traits (e.g., host adaptation, climate change resilience, prevalence under changing soil and cropping systems, increased sporulation) for long-term persistence in a target area (Mehl et al., 2012). Communities dominated by one or a few VCGs may not be stable over the long-term (Ortega-Beltran and Cotty, 2018) and therefore rotation of multi-genotype biocontrol products/AAVs could be beneficial. Rotating Aflasafe GH01 and Aflasafe GH02 in Ghana could serve to test if a more robust aflatoxin control strategy is achieved with more complex atoxigenic AAV communities.

The atoxigenic VCGs used in the current study are native to and widely-distributed in Ghana. Large-scale use of either Aflasafe GH01 or Aflasafe GH02 throughout Ghana can help farmers produce crops with greatly reduced aflatoxin content, thereby reducing dietary exposure and concomitant health effects while improving trade opportunities and income of the Ghanaian people. Current activities in Ghana include characterization of obstacles to large-scale adoption of biocontrol use and development of protocols to circumvent those obstacles. Ultimately, large-scale use of these biocontrol products, which solve an invisible problem, will result when appropriate technological, social, and institutional approaches converge into a holistic approach to address the frequent detriment of crop aflatoxin contamination in Ghana.

## CRediT authorship contribution statement

**Daniel Agbetiameh:** Methodology, Validation, Formal analysis, Investigation, Resources, Writing - original draft, Data curation, Writing - review & editing. **Alejandro Ortega-Beltran:** Validation, Formal analysis, Data curation, Writing - review & editing. **Richard T. Awuah:** Conceptualization, Methodology, Writing - review & editing, Supervision. **Joseph Atehnkeng:** Conceptualization, Methodology, Formal analysis, Writing - review & editing. **Abuelgasim Elzein:** Methodology, Validation, Formal analysis, Investigation, Data curation, Writing - review & editing. **Peter J. Cotty:** Conceptualization, Methodology, Writing - review & editing, Funding acquisition. **Ranajit Bandyopadhyay:** Conceptualization, Methodology, Investigation, Resources, Writing - review & editing, Supervision, Project administration, Funding acquisition.

## Declaration of Competing Interest

The authors receive no direct financial benefit from the biocontrol technology described in this work. Initial patents for use of atoxigenic strains to prevent aflatoxin contamination were filed in 1988 and awarded by the US patent office to the US Department of Agriculture in 1992 and 1994 with Peter Cotty as the inventor. The patent protection has expired. The manufacturing process for and the nature of compositions of Aflasafe GH01 and Aflasafe GH02, the biocontrol products discussed in the current work, are not patented and are used for several atoxigenic strain-based products that differ primarily in the active ingredient genotypes based on counry of deployment. In addition to the Ghana Aflasafe products, these products include other products bearing the Aflasafe name (e.g., Aflasafe, Aflasafe SN01, Aflasafe KE01, etc.), AF36 Prevail in the US, and AF X-1 in Italy (Mauro et al., 2015; Bandyopadhyay et al., 2016; Ortega-Beltran and Bandyopadhyay, 2019; Senghor et al., 2020). The fungal isolates used as active ingredients of Aflasafe GH01 and Aflasafe GH02 are considered a portion of the bioresources of Ghana and, as such, are not patented. However, atoxigenic genotypes suitable for biocontrol applications have been found in all regions where active ingredients have been sought (Bandyopadhyay et al., 2016). The Aflasafe name is a Trademark of the International Institute of Tropical Agriculture (IITA). During 2019, IITA manufactured Aflasafe GH02 and gave temporary rights for distribution in Ghana to private companies. Manufacturing and distribution of Aflasafe products in Ghana is expected to be transferred to the private sector during 2020. IITA will charge a licensing fee to the manufacturer for use of the Aflasafe name and associated technology transfer. Ranajit Bandyopadhyay, Alejandro Ortega-Beltran, and Joseph Atehnkeng are currently employed by IITA.
