# Supplementary material for: Field efficacy of two atoxigenic biocontrol products for mitigation of aflatoxin contamination in maize and groundnut in Ghana
Source: Biol Control. 2020 Nov;150:104351. doi: 10.1016/j.biocontrol.2020.104351 (PMC7457722; doi:10.1016/j.biocontrol.2020.104351)
Supplement: Supplementary data 2 [file mmc2.docx]

Supplementary Table 1. Combined frequencies and distribution of Aspergillus section Flavi in soils and grains from Aflasafe GH01 treated and untreated fields in five regions across three agroecological zones (AEZs) in Ghana.

| Year | AEZ^a^ | Region | Treatment^b^ | Frequencies of *Aspergillus* section *Flavi*^c,d^ (%) | | | | | | | | | | | | | |
| --- | --- | --- | --- | --- | --- | --- | --- | --- | --- | --- | --- | --- | --- | --- | --- | --- | --- |
|  |  |  |  | Soil before inoculation | | | |  | Soil at harvest | | | |  | Grains^e^ | | | |
|  |  |  |  | L | S_BG_ | P | T |  | L | S_BG_ | P | T |  | L | S_BG_ | P | T |
| 2015 | DS | Brong Ahafo | Treated | 96.9 | 0.0 | 0.0 | 3.2 |  | 98.9 | 0.0 | 1.2 | 0.0 |  | 100.0 | 0.0 | 0.0 | 0.0 |
|  |  |  | Untreated | 94.8 | 1.1 | 1.1 | 3.2 |  | 91.7 | 3.2 | 3.1 | 2.1 |  | 97.9 | 2.1 | 0.0 | 0.0 |
|  |  | Northern | Treated | 98.9 | 0.0 | 0.6 | 0.6 |  | 99.5^*^ | 0.0^*^ | 0.0 | 0.6 |  | 98.5 | 0.0 | 0.0 | 1.6 |
|  |  |  | Untreated | 94.8 | 2.1 | 1.1 | 2.1 |  | 88.0 | 11.5 | 0.0 | 0.6 |  | 99.4 | 0.0 | 0.7 | 0.0 |
|  | HF | Ashanti | Treated | 83.4 | 0.0 | 0.0 | 16.7^*^ |  | 93.7 | 0.6 | 0.0 | 5.8 |  | 100.0 | 0.0 | 0.0 | 0.0 |
|  |  |  | Untreated | 90.7 | 4.7 | 1.0 | 3.7 |  | 89.1 | 0.0 | 1.1 | 9.9 |  | 100.0 | 0.0 | 0.0 | 0.0 |
|  |  | Brong Ahafo | Treated | 85.4 | 0.0 | 11.5 | 3.2 |  | 100.0^*^ | 0.0 | 0.0^*^ | 0.0 |  | 100.0 | 0.0 | 0.0 | 0.0 |
|  |  |  | Untreated | 79.8 | 1.1 | 18.2 | 1.1 |  | 83.4 | 1.1 | 14.6 | 1.1 |  | 96.9 | 3.2 | 0.0 | 0.0 |
|  | SGS | Upper East | Treated | 97.9 | 0.0 | 0.0 | 2.1 |  | 98.9 | 0.0 | 0.6 | 0.6 |  | 98.5 | 0.0 | 0.0 | 1.6 |
|  |  |  | Untreated | 98.5 | 1.1 | 0.0 | 0.5 |  | 96.8 | 2.1 | 0.6 | 0.5 |  | 99.5 | 0.0 | 0.0 | 0.6 |
|  |  | Upper West | Treated | 95.0 | 0.0 | 3.5 | 1.6 |  | 98.5 | 0.0 | 1.6 | 0.0 |  | 100.0 | 0.0 | 0.0 | 0.0 |
|  |  |  | Untreated | 93.8 | 2.6 | 1.1 | 2.6 |  | 97.9 | 0.0 | 1.6 | 0.6 |  | 98.2 | 1.3 | 0.0 | 0.6 |
|  |  |  |  |  |  |  |  |  |  |  |  |  |  |  |  |  |  |
| 2016 | DS | Brong Ahafo | Treated | 89.9 | 5.2 | 1.0 | 4.0 |  | 99.0 | 0.0 | 0.0 | 1.0 |  | 100.0 | 0.0 | 0.0 | 0.0 |
|  |  |  | Untreated | 88.7 | 1.0 | 9.3 | 1.0 |  | 92.9 | 4.2 | 1.0 | 2.0 |  | 97.9 | 2.2 | 0.0 | 0.0 |
|  |  | Northern | Treated | 96.5 | 0.0 | 3.0 | 0.5 |  | 99.5 | 0.0 | 0.0 | 0.5 |  | 100.0 | 0.0 | 0.0 | 0.0 |
|  |  |  | Untreated | 91.2 | 1.6 | 0.5 | 6.8 |  | 90.1 | 5.8 | 1.6 | 2.6 |  | 99.5 | 0.5 | 0.0 | 0.0 |
|  | HF | Ashanti | Treated | 97.4 | 1.1 | 0.5 | 1.0 |  | 99.0 | 0.5 | 0.0 | 0.5 |  | 100.0 | 0.0 | 0.0 | 0.0 |
|  |  |  | Untreated | 96.4 | 0.5 | 3.1 | 0.0 |  | 94.4 | 1.6 | 1.1 | 3.1 |  | 98.0 | 1.0 | 0.5 | 0.5 |
|  |  | Brong Ahafo | Treated | 85.4 | 4.2 | 4.2 | 6.4 |  | 94.9 | 0.0 | 0.0 | 5.2 |  | 100.0 | 0.0 | 0.0 | 0.0 |
|  |  |  | Untreated | 94.9 | 1.0 | 3.2 | 1.0 |  | 99.0 | 1.0 | 0.0 | 0.0 |  | 100.0 | 0.0 | 0.0 | 0.0 |
|  | SGS | Upper East | Treated | 98.5 | 0.0 | 1.5 | 0.0 |  | 100.0 | 0.0 | 0.0 | 0.0 |  | 98.0 | 2.0 | 0.0 | 0.0 |
|  |  |  | Untreated | 90.4 | 2.5 | 0.0 | 7.2 |  | 98.5 | 0.0 | 1.5 | 0.0 |  | 94.9 | 2.2 | 2.0 | 1.0 |
|  |  | Upper West | Treated | 84.6 | 3.8 | 3.3 | 8.4 |  | 100.0 | 0.0 | 0.0 | 0.0 |  | 98.5^*^ | 0.0 | 0.0^*^ | 1.5 |
|  |  |  | Untreated | 89.3 | 3.3 | 0.8 | 6.8 |  | 94.0 | 0.8 | 0.0 | 5.3 |  | 86.0 | 0.0 | 14.0 | 0.0 |

^a^ DS, Derived Savanna; HF, Humid Forest; SGS, Southern Guinea Savanna.

^b^ Treated refers to fields to which Aflasafe GH01 was applied at the rate of 10 kg/ha. Untreated were nearby fields separated by at least 25 m from corresponding treated field in which no biocontrol product was applied.

^c^ L = *A. flavus* L morphotype, S_BG_ = S_BG_ strains, P = *A. parasiticus,* T = *A. tamarii.*

^e^ In each region, species frequencies from treated samples with an asterisk (*) significantly differed from those found in corresponding untreated samples by Student’s *t*-test (α = 0.05).

^e^ Values depict means for both maize and groundnut grains.
