# Supplementary material for: Field efficacy of two atoxigenic biocontrol products for mitigation of aflatoxin contamination in maize and groundnut in Ghana
Source: Biol Control. 2020 Nov;150:104351. doi: 10.1016/j.biocontrol.2020.104351 (PMC7457722; doi:10.1016/j.biocontrol.2020.104351)
Supplement: Supplementary data 3 [file mmc3.docx]

Supplementary Table 2. Combined frequencies and distribution of *Aspergillus* section Flavi in soils and grains collected from Aflasafe GH02 treated and untreated fields in five regions across three agroecological zones (AEZs) in Ghana.

| Year | AEZ^a^ | Region | Treatment^b^ | Frequencies of *Aspergillus* section *Flavi*^c,d^ (%) | | | | | | | | | | | | | |
| --- | --- | --- | --- | --- | --- | --- | --- | --- | --- | --- | --- | --- | --- | --- | --- | --- | --- |
|  |  |  |  | Soil before inoculation | | | |  | Soil at harvest | | | |  | Grains^e^ | | | |
|  |  |  |  | L | S_BG_ | P | T |  | L | S_BG_ | P | T |  | L | S_BG_ | P | T |
| 2015 | DS | Brong Ahafo | Treated | 79.2 | 0.0 | 16.7 | 4.2 |  | 97.0^*^ | 1.0 | 0.0^*^ | 2.0 |  | 97.9 | 2.1 | 0.0 | 0.0 |
|  |  |  | Untreated | 87.5 | 2.1 | 8.4 | 2.1 |  | 68.7 | 5.2 | 26.2 | 0.0 |  | 95.9 | 4.2 | 0.0 | 0.0 |
|  |  | Northern | Treated | 99.5 | 0.0 | 0.0 | 0.6 |  | 100.0 | 0.0 | 0.0 | 0.0 |  | 100.0 | 0.0 | 0.0 | 0.0 |
|  |  |  | Untreated | 98.4 | 0.6 | 1.1 | 0.0 |  | 94.3 | 4.2 | 0.6 | 1.1 |  | 98.9 | 0.6 | 0.0 | 0.6 |
|  | HF | Ashanti | Treated | 86.5 | 1.1 | 9.4 | 3.1 |  | 92.2 | 2.6 | 0.6 | 4.7 |  | 100.0^*^ | 0.0^*^ | 0.0 | 0.0 |
|  |  |  | Untreated | 83.9 | 1.6 | 6.3 | 8.3 |  | 93.3 | 4.1 | 0.0 | 2.7 |  | 76.1 | 24.0 | 0.0 | 0.0 |
|  |  | Brong Ahafo | Treated | 71.5 | 0.0 | 28.5 | 0.0 |  | 97.9^*^ | 1.1 | 1.1^*^ | 0.0 |  | 99.0 | 1.1 | 0.0 | 0.0 |
|  |  |  | Untreated | 62.5 | 3.2 | 34.4 | 0.0 |  | 83.4 | 0.0 | 16.7 | 0.0 |  | 95.9 | 4.2 | 0.0 | 0.0 |
|  | SGS | Upper East | Treated | 100.0 | 0.0 | 0.0 | 0.0 |  | 97.9 | 1.6 | 0.0 | 0.6 |  | 100.0 | 0.0 | 0.0 | 0.0 |
|  |  |  | Untreated | 95.9 | 0.5 | 1.6 | 2.1 |  | 98.2 | 1.8 | 0.0 | 0.0 |  | 100.0 | 0.0 | 0.0 | 0.0 |
|  |  | Upper West | Treated | 96.4 | 0.0 | 0.0 | 3.7 |  | 99.5 | 0.0 | 0.0 | 0.6 |  | 100.0 | 0.0 | 0.0 | 0.0 |
|  |  |  | Untreated | 96.9 | 1.1 | 0.0 | 2.1 |  | 95.3 | 0.0 | 1.6 | 3.2 |  | 99.0 | 0.0 | 0.0 | 1.1 |
|  |  |  |  |  |  |  |  |  |  |  |  |  |  |  |  |  |  |
| 2016 | DS | Brong Ahafo | Treated | 95.9 | 1.1 | 0.0 | 3.1 |  | 100.0 | 0.0 | 0.0 | 0.0 |  | 99.0 | 1.1 | 0.0 | 0.0 |
|  |  |  | Untreated | 95.8 | 1.1 | 1.1 | 2.1 |  | 88.6 | 1.1 | 5.2 | 5.2 |  | 99.0 | 1.1 | 0.0 | 0.0 |
|  |  | Northern | Treated | 89.6 | 0.0 | 0.0 | 10.5 |  | 100.0 | 0.0 | 0.0 | 0.0 |  | 98.5 | 0.0 | 0.0 | 1.6 |
|  |  |  | Untreated | 93.2 | 1.6 | 3.2 | 2.1 |  | 90.1 | 4.2 | 0.0 | 5.8 |  | 99.0 | 0.0 | 1.1 | 0.0 |
|  | HF | Ashanti | Treated | 89.6 | 3.7 | 2.6 | 4.2 |  | 99.0 | 0.0 | 0.0 | 1.1 |  | 97.9 | 1.1 | 1.1 | 0.0 |
|  |  |  | Untreated | 95.9 | 0.6 | 2.6 | 1.0 |  | 99.5 | 0.6 | 0.0 | 0.0 |  | 93.2 | 5.8 | 0.0 | 1.1 |
|  |  | Brong Ahafo | Treated | 100.0 | 0.0 | 0.0 | 0.0 |  | 99.0 | 0.0 | 1.1 | 0.0 |  | 99.0 | 0.0 | 1.1 | 0.0 |
|  |  |  | Untreated | 89.6 | 1.1 | 7.3 | 2.1 |  | 90.6 | 3.2 | 5.2 | 1.1 |  | 100.0 | 0.0 | 0.0 | 0.0 |
|  | SGS | Upper East | Treated | 95.3 | 1.6 | 0.0 | 3.2 |  | 100.0 | 0.0 | 0.0 | 0.0 |  | 100.0 | 0.0 | 0.0 | 0.0 |
|  |  |  | Untreated | 98.5 | 1.6 | 0.0 | 0.0 |  | 92.2 | 3.2 | 3.1 | 1.6 |  | 100.0 | 0.0 | 0.0 | 0.0 |
|  |  | Upper West | Treated | 97.4 | 0.6 | 0.5 | 1.6 |  | 100.0^*^ | 0.0^*^ | 0.0 | 0.0 |  | 100.0 | 0.0 | 0.0 | 0.0 |
|  |  |  | Untreated | 91.2 | 0.5 | 7.3 | 1.1 |  | 75.5 | 21.9 | 1.1 | 1.6 |  | 99.5 | 0.0 | 0.6 | 0.0 |

^a^ DS, Derived Savanna; HF, Humid Forest; SGS, Southern Guinea Savanna.

^b^ Treated refers to fields to which Aflasafe GH01 was applied at the rate of 10 kg/ha. Untreated were nearby fields separated by at least 25 m from corresponding treated field in which no biocontrol product was applied.

^c^ L = *A. flavus* L morphotype, S_BG_ = S_BG_ strains, P = *A. parasiticus,* T = *A. tamarii.*

^d^ In each region, species frequencies from treated samples with an asterisk (*) significantly differed from those found in corresponding untreated samples by Student’s *t*-test (α = 0.05).

^e^ Values depict means for both maize and groundnut grains.
